# Tunable sulphur doping on CuFe_2_O_4_ nanostructures for the selective elimination of organic dyes from water

**DOI:** 10.1038/s41598-023-33185-0

**Published:** 2023-04-18

**Authors:** Anam Aslam, Muhammad Zeeshan Abid, Khezina Rafiq, Abdul Rauf, Ejaz Hussain

**Affiliations:** Institute of Chemistry, Inorganic Materials Laboratory 52S, The Islamia University of Bahawalpur, Bahawalpur, 63100 Pakistan

**Keywords:** Chemistry, Materials science, Nanoscience and technology

## Abstract

In this work, sulphur doped copper ferrites (S-CuFe_2_O_4_) photocatalysts were successfully synthesized for the first time using the facile hydrothermal method. The as-synthesized photocatalysts were characterized through XRD, Raman, TGA, FT-IR, UV–Vis-DRS, SEM, EDX and PL techniques. The results revealed that doping with sulphur has been found to be a suitable alternative that causes strain in the lattices as anions replace the oxygen from the CuFe_2_O_4_ nanostructures. Due to sulphur dopants, photocatalysts are able to efficiently trap and transfer the photoinduced charges, which readily suppress charge recombination. A UV–Vis spectrophotometer was used to monitor the degradation of selective toxic organic dyes (RhB, CR, MO, and CV) in aqueous media. The dye degradation results provide evidence for the surprisingly superior performance of S-CuFe_2_O_4_ over pristine CuFe_2_O_4_. On the basis of its efficiencies, this work can be assigned as an excellent candidate for photocatalysis science.

## Introduction

Water pollution is one of the world's most serious environmental problems. Industrialization produces wastewater that contains a variety of harmful pollutants^1^. The most prevalent pollutants are synthetic organic dyes, which are widely used in variety of industries, and pose severe threats to the aquatic environment^2^. Azo dyes that are widely used in textile industries are proven to be carcinogenic and killers for liver cells^3^. Similarly, methyl orange (MO) and crystal violet (CV) are toxic, water-soluble anionic azo dyes with complex chemical structures that cause gastrointestinal, respiratory, and skin irritations. Congo red (CR) is an azo dye that is carcinogenic, mutagenic, and causes reproductive problems^4^. Rhodamine B (RhB) is a fluorescein dye that causes skin and eye irritation and is toxic if swallowed. These fluorescein dyes are extremely cytotoxic to mammalian tissues and cause morphological and genetic changes^5,6^. Thus, polluted water is becoming a challenge, especially for the urban communities lying near industrial zones. Therefore, it is crucial to remove these dyes from wastewater using an economical approach^7,8^.

Various strategies have been developed for the degradation of dyes in contaminated water. Adsorption, electrochemical deposition, redox reactions and biological treatment are examples of these methods^9,10^. However, due to the inherent toxicity, production of secondary pollutants and high cost of these approaches, these methods proved ineffective^11^. More research is obligatory to develop cost-effective, stable and risk-free materials to address these issues. Photocatalytic dye degradation is a low-cost, versatile and energy-efficient process that breaks down organic dyes in water using light and a catalyst^12,13^. For this purpose, various metal oxides, sulphides, quantum dots, noble metal nanoparticles, polymers, metal and non-metal doped hybrid materials, graphene-based materials, gold nanoclusters and transition metal ferrites have been used^14–24^. Among these, nanomaterials of transition metal ferrites have low cost, high chemical stability, a large surface area, reusability and catalytic properties^25^.

For dye degradation applications, a number of ferrites including spinel ferrites, have been reported to be used as catalysts^26^. Thus, spinel ferrites are the type M^2+^M_2_^3+^O_4_ class of compounds that have attracted great attention for dye degradation studies^27,28^. The typical formula for spinel ferrites is MFe_2_O_4_, where M = Zn, Mn, Ni, Cu, etc. The composition and structure of ferrites influence their ability to adsorb substances, which is dependent on their morphology and inherent crystal structure^29^. CuFe_2_O_4_ is a spinel-type material with a narrow band gap, photochemical stability and visible light activity^28^. Moreover, its low toxicity, cost-effectiveness, versatility, and recyclability make it an attractive catalyst for water purification applications^30,31^. Various methods have been reported to synthesize spinel ferrites microstructures such as sol–gel, photo as well as electro deposition, solid state reaction, hydrothermal method and coprecipitation^32^. Hydrothermal method is preferred due to versatility, uniformity, purity and ease of synthesis^33^.

However, due to the rapid recombination of photo-generated charge carriers, it is necessary to improve the effectiveness of CuFe_2_O_4_^34^. To improve photocatalytic efficiencies, a variety of techniques, such as construction of nanocomposite^35^, p-n junctions^36^ and metals and non-metals doping^37^ have been used. Selective doping can recast the band structure by generating quasi-stable energies^38^. Recently, sulphur dopants on g-C_3_N_4_, graphene sheets^39,40^ and ZnO have been reported to improve photocatalytic applications^41^. Moreover, because of the significant difference in size and electronegativity between S and O, S dopants modifies the optical, electrical and photocatalytic properties of semiconductor oxide^42^.

To modify the conventional ferrites and acquire the advantage of S-dopants, the hydrothermal method was adopted to synthesize S-doped copper ferrite (S-CuFe_2_O_4_) photocatalysts. The as-prepared CuFe_2_O_4_ and S-CuFe_2_O_4_ catalysts were used for degradation of the selective dyes like rhodamine B (RhB), congo red (CR), crystal violet (CV) and methyl orange (MO) as a probe reaction. The results demonstrate that S-CuFe_2_O_4_ photocatalysts demonstrate higher photocatalytic efficiencies as compared to pristine CuFe_2_O_4._

## Experimental

### Catalyst preparation

**Figure 1:**
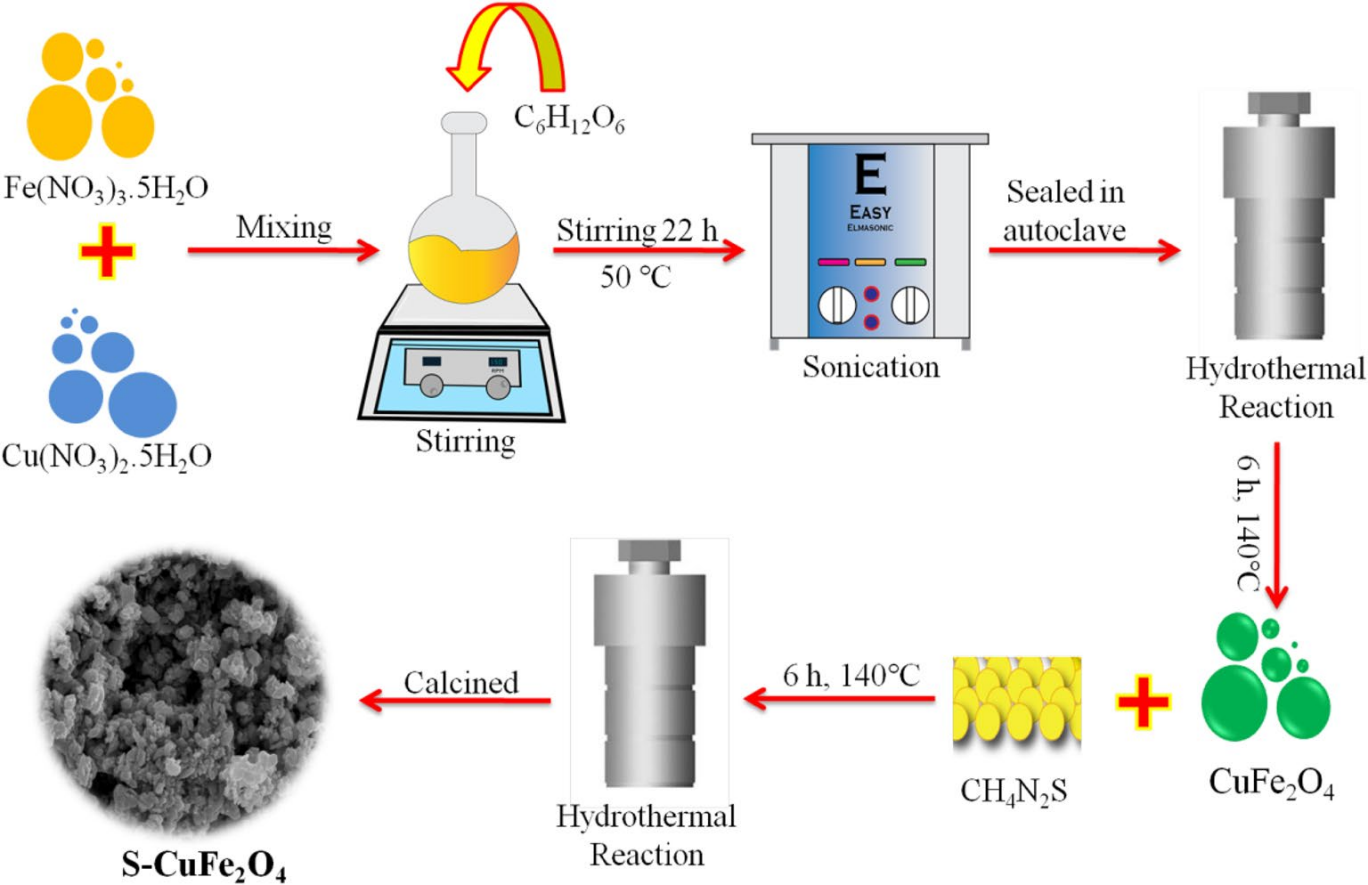
Scheme used for the synthesis of CuFe_2_O_4_ and S-CuFe_2_O_4_.

Copper ferrites were synthesized by hydrothermal method as follows: Solutions of 0.1 M copper nitrate and 0.2 M iron nitrate magnetic stirred for 30 min. After that, 0.01 M glucose solution was transfer to above solution and left it for stirring (22 h, 50 °C), the preparations of solutions were explained in (supporting information) section. The obtained mixture solution was then sonicated for 30 min by adjusting the sweep frequency (≈ 37 kHz, 300W). The final solution was kept in autoclave (Sanfa, DHG-9030) for 6 h at 140 °C, which is then cooled down and filtered by using high grade filter paper (WHA 1001325, Grade-1). The obtained precipitates were washed thoroughly using deionized water and then with absolute ethanol. To prepare sulphur doped copper ferrites (S-CuFe_2_O_4_), 500 mg of CuFe_2_O_4_ powder was transferred to autoclave reactor and appropriate amount of 1% thiourea solution (5 mg, 0.01 M) was added into this mixture and mixture was again kept in autoclave for 6 h at 140 °C. The synthesis scheme was illustrated in Fig. 1.

### Characterization

To predict the morphology and crystal structures of S-CuFe_2_O_4,_ powder X-ray diffraction (XRD) results were obtained at Philips-X Pertpro, with Cu kα radiation, λ = 1.5406 Å. For the surface morphology of as-prepared catalysts, SEM analyses were conducted on a Field Emission Scanning Electron microscope (FEI-Nova Nano SEM-450). Energy dispersive X-ray analysis (EDX, Horiba 7593 H) afforded the chemical composition of the prepared catalysts. The ultraviolet–visible-diffuse reflectance (UV–Vis-DRS) results were obtained at AvaSpec-2048 TEC spectrometer. The Photoluminescence (PL) results were recorded using Fluorescence spectrometer (LS-45, Perkin Elmer), where PL intensities were measured at 300–650 nm range. Dye degradation experiments were performed using UV–Vis spectrophotometer (PerkinElmer/λ-365. TGA was performed using TG209 F3Tarsus^®^, NETZSCH Germany. Fourier Transform Infra-Red (FT-IR) analyses were performed on Bruker Tensor-27. Raman analysis was done by using JASCO NRS-5000/7000 dispersive Raman spectrometers.

### Photocatalytic reactions

The photocatalytic degradation properties of pristine CuFe_2_O_4 _and S-CuFe_2_O_4_ were investigated in the presence of visible-light radiations within Pyrex reactor. The photoreactor was cylindrical, having a 500 mL volume capacity with an 8 cm internal and 10 cm external diameter. In order to prevent light emission into the surrounding environment, the outer wall of the reactor was covered by aluminum foil. The cool water was allowed to circulate around the walls of the reactor in order to keep it at room temperature. A halogen lamp having λ ˃ 400 nm was used as a radiation source. The 10 mg amount of photocatalyst was fixed for each photoreaction at pH 7 for 15 ppm dye solution (vol = 100 mL). Prior to photoreaction, the solutions were stored in the dark (absence of light) to ensure the adsorption equilibrium. For the photoreaction, a halogen lamp was fixed at a distance of 30 cm from the reactor. The photocatalytic degradation efficiency was examined at different time intervals using a spectrophotometer (PerkinElmer/λ-365).

## Results and discussion

The synthesis procedure of our S-CuFe_2_O_4_ photocatalysts is shown in Fig. 1 (see details in the “Experimental” section). To remove the impurities and enhance the crystallinity, the as-synthesized photocatalysts were calcined at 350 °C for 3 h.

### XRD

**Figure 2:**
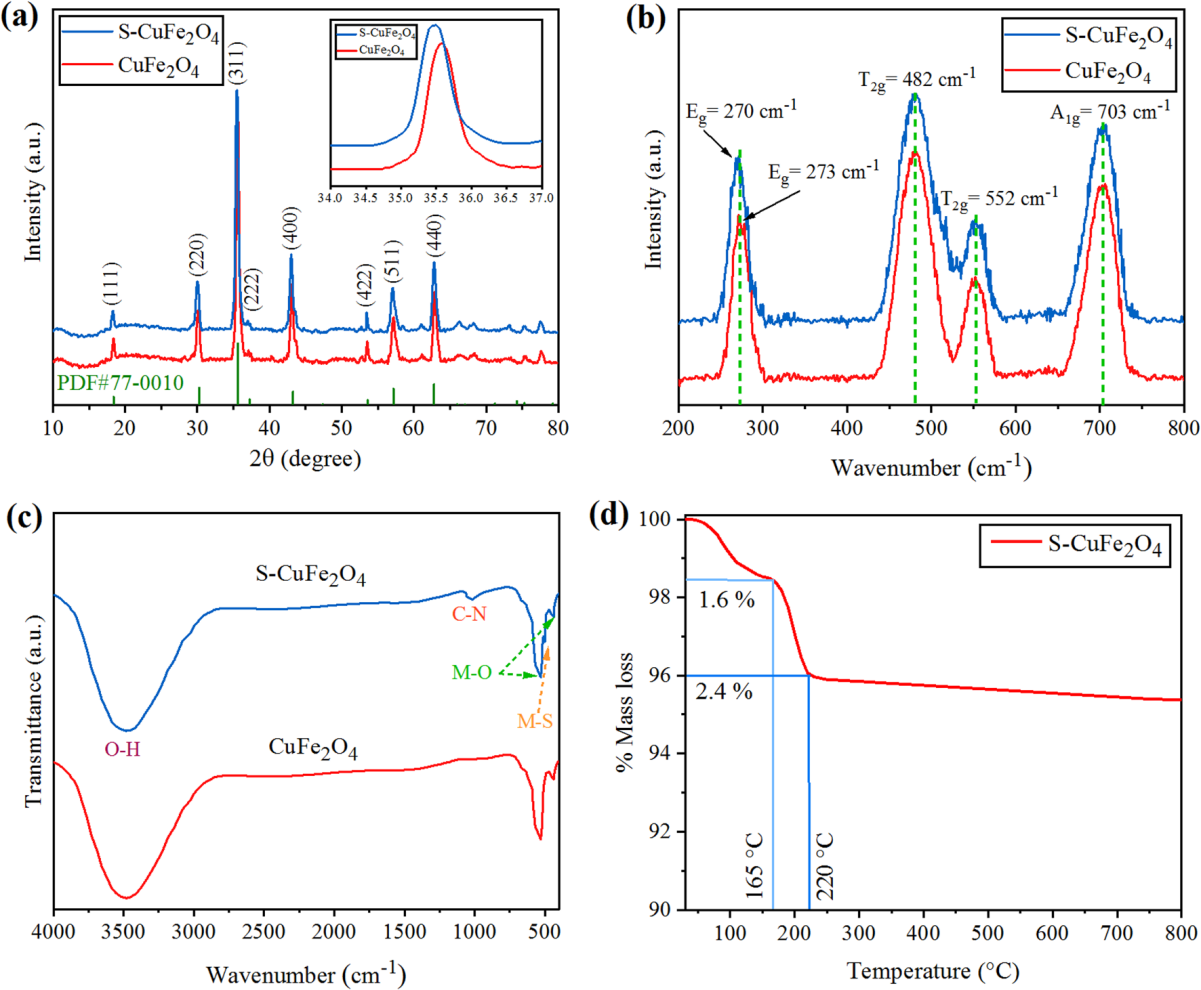
(**a**) XRD patterns exhibited with zoomed image in the inset, (**b**) Raman spectrum, (**c**) FT-IR spectrum of CuFe_2_O_4_ and S-CuFe_2_O_4_, (**d**) TGA of S-CuFe_2_O_4_ photocatalyts.

The phase purity and crystalline nature of as-prepared product was determined by XRD. The XRD pattern of copper ferrite nanostructures is shown in Fig. 2a. The XRD patterns exactly match the standard JCPDS file No. 77-0010^43^. The diffraction peaks of as-synthesized catalysts are located at 18.34°, 30.17°, 35.54°, 37.18°, 43.19°, 53.59°, 57.13° and 62.744° that are associated with Millar indices as (111), (220), (311), (222), (400), (422), (511) and (440) respectively. These XRD patterns indicate that as-synthesized catalysts emphasize cubic spinel structures, where Fe^+3^ ions occupy octahedral while Cu^+2^ ions occupy tetrahedral sites. It has been noted that due to sulphur doping major peaks shifts to the lower 2 theta whereas intensity of diffraction peaks has been observed to be decreased as well. The XRD results depict that the replacement of larger sulphur anions was accomplished with the smaller oxygen ions in the CuFe_2_O_4_ structure. No other crystalline phases of Fe_2_O_3_, FeS or CuS were detected that confirm the purity of the catalysts, some extra peaks beyond 65° were observed due to some CuO contents^44^. The sharpness of peaks indicates that the designed catalysts of this work are highly crystalline in nature. The crystalline sizes of pure CuFe_2_O_4_ and S-CuFe_2_O_4_ have been calculated by using Debye‒Scherrer’s Formula in Eq. (1).

1 $$\mathrm{D}=\frac{0.94\uplambda }{{\upbeta \cos \uptheta }}$$

where λ is 1.54060 Å, β is FWHM and θ is diffraction angle (Cu‒Kα). The Williamson‒Hall method was also used to calculate the crystallite size of both pure CuFe_2_O_4_ and S-CuFe_2_O_4_ Eq. (2).

2 $${\upbeta \cos \uptheta }= \frac{K\lambda }{d} + 2\,{\varepsilon \sin \uptheta }$$

where d is crystalline size, K is constant = 0.94, ε is constant = 1^45^. The crystallite size (D) was calculated using the Scherer formula and Williamson‒Hall method are tabulated in Table 1, whereas, the Williamson-Hall plots are depicted in Fig. S1.

**Table 1:** Comparison of crystallite sizes and band gap energies of as-synthesized photocatalysts.

Sr. no.	Catalyst name	Crystal size Scherer method (nm)	Crystal size W–H method (nm)	Band gap (eV)
1	CuFe_2_O_4_	17.85	22.73	1.80
2	S-CuFe_2_O_4_	18.54	24.01	1.78

### Raman spectroscopy

Raman spectroscopy is an efficient technique for direct probe of structural transformation, cation redistribution, bonding in metal oxides and lattice distortion^30^. Figure 2b represents Raman spectra of CuFe_2_O_4_ and S-CuFe_2_O_4_. Group theory analysis yields four Raman active modes denoted by *A*_1g_, *E*_g_ and two *T*_2g_ modes in range of 200–800 cm^−1^^31^. The *A*_1g_ mode observed at ∼ 703 cm^−1^, *E*_g_ mode at ∼ 273 cm^−1^, the two *T*_2g_ modes ∼ 482 cm^−1^ and ∼ 552 cm^−1^. The obtained Raman frequency relies on the Fe(Cu)–O bond length, that varies with the major factors, like phase transformation, lattice distortion and cationic redistribution^32^. It has been noted that sulphur dopants in copper ferrite shift the *E*_g_ mode slightly to a smaller wave number (273 to 270 cm^−1^). Lattice distortion was observed, between two *T*_2g_ modes ascribed majorly due to the sulphur dopants as compared to oxygen contents present in catalysts^33^.

### FT-IR

The Fourier transforms infrared spectrum of CuFe_2_O_4_ and S-CuFe_2_O_4_ nanoparticles are shown in Fig. 2c. In the present work, major FTIR peak observed at 530 cm^−1 ^is due to M–O vibration of tetrahedron where M represents copper or iron. Similarly, peak observed at 436 cm^−1^ was referred to the stretching of octahedron planes of spinel copper oxides. The broad band located at around 3500 cm^−1^ are related to bending vibration of O–H, which correspond to the hydroxyl groups or absorbed water molecules over the catalyst’s surfaces. It is obvious that upon doping sulphur content, the metal sulphur vibrations lies at 500 cm^−1^, exhibit low intensity than that of the Cu–O/Fe–O vibration peaks. FTIR patterns located approximately at 1020–1030 cm^−1^ is due to the C–N stretching, which are likely to be caused by thiourea^46^. These stretching vibrations are also observed by other researchers working on S dopants^47^. These nitrogen/carbon (N–C) vibrations are absent in the FT-IR spectra of pristine CuFe_2_O_4_.

### Thermo gravimetric analysis (TGA)

Thermo gravimetric analysis of as-synthesized S-CuFe_2_O_4_ (without calcination) was performed to investigate physical behavioral changes (Fig. 2d). These evaluations were performed under N_2_ atmosphere; within the temperature range 35–800 °C where increment in heating rate was maintained 5 °C/min. It has been observed that 1.6% weight loss was observed up to 165 °C, this loss can be attributed to the volatilization of absorbed water and solvent molecules^48^. Moreover, further 2.4% weight loss up to 220 °C was observed, endorsed to decomposition of metal hydroxide^48,49^. Afterwards, by increasing the temperature up to 800 °C no weight loss was observed, this exhibited excellent thermal stability of S-CuFe_2_O_4_ nanoparticles.

### SEM with EDX analysis

**Figure 3:**
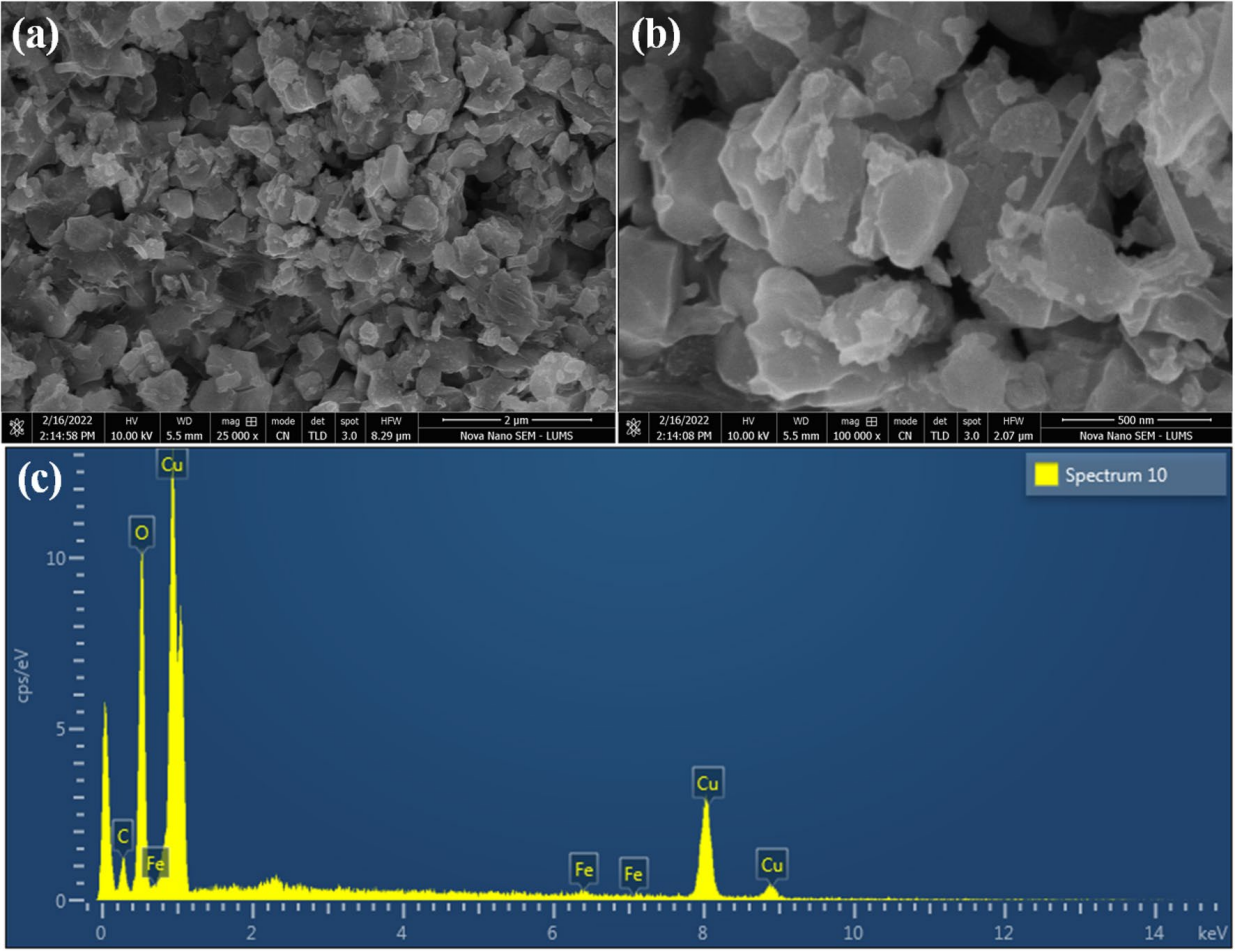
SEM images of pristine CuFe_2_O_4_ at (**a**) 2 μm, (**b**) 500 nm, (**c**) represents the EDX analysis.

**Figure 4:**
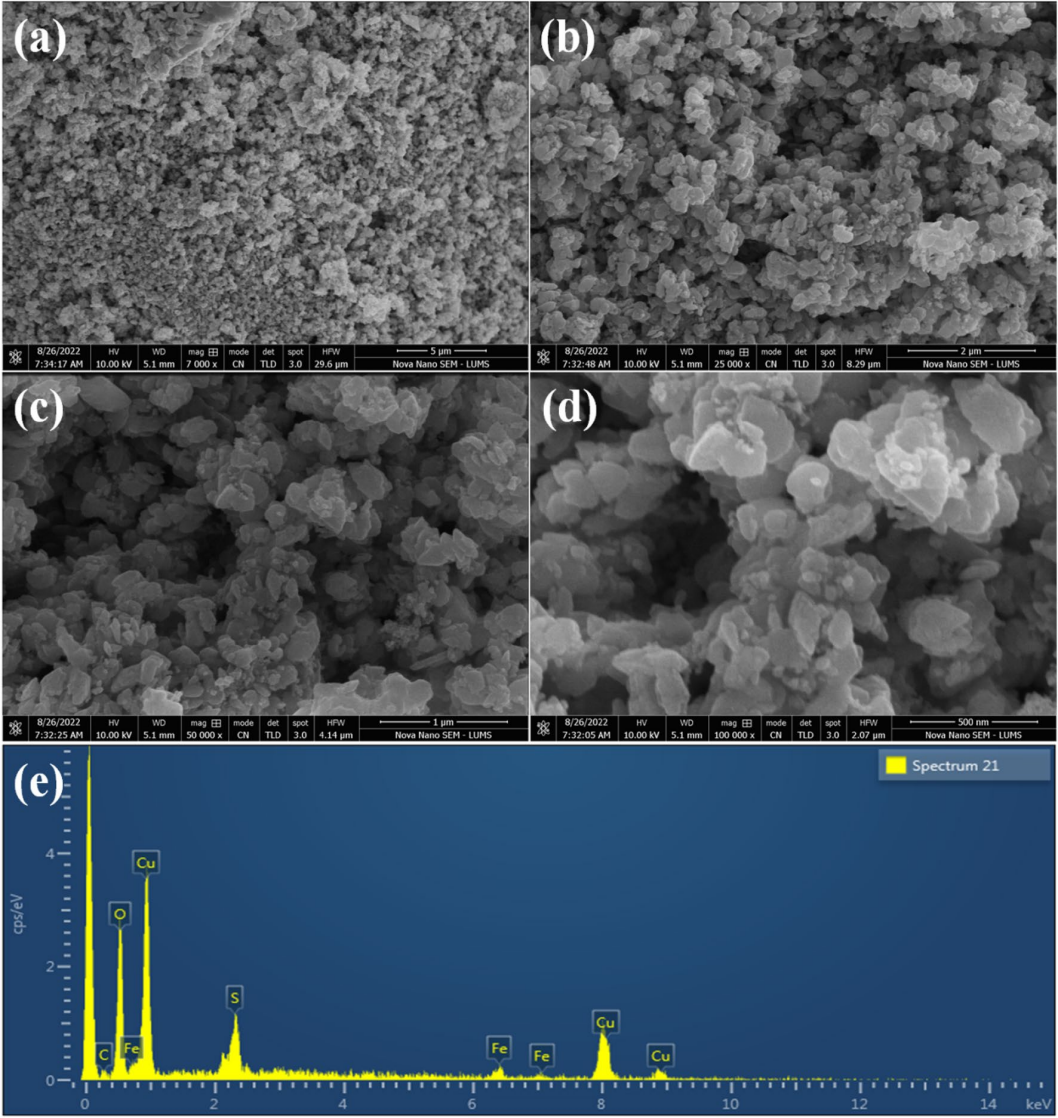
SEM images of S-CuFe_2_O_4_ at (**a**) 5 μm, (**b**) 2 μm, (**c**) 1 μm and (**d**) 500 nm, whereas (**e**) represents the EDX analysis.

The morphologies of pristine CuFe_2_O_4_ and S-CuFe_2_O_4_ nanostructures were investigated by SEM (equipped with ETD/TLD detector). The SEM images of pristine CuFe_2_O_4_ are demonstrated in Fig. 3a,b. The results show that the particles are agglomerated and have irregular flacks-like morphologies. The particles have a tendency to agglomerate in clusters due to the attractive forces (i.e., magnetic dipole–dipole)^50^. Whereas, the SEM results of as-prepared S-CuFe_2_O_4_ catalysts are shown in Fig. 4a–d at different magnifications (i.e., 5 µm, 2 µm, 1 µm and 500 nm). It is clear from SEM results that by doping with sulphur, the flakes-like morphology of catalysts became more regular and agglomeration was reduced, which indicate the porosity of catalysts^51^. Thus, more active sites are available for photocatalytic reactions. These results depict that S-doping enhances the surface area and assist to increase the catalytic activity of CuFe_2_O_4_. EDX analyses provide valuable information on the composition and distribution of elements. The EDX results of as-synthesized pristine CuFe_2_O_4_ and S-CuFe_2_O_4_ photocatalysts are illustrated in Figs. 3c and 4e, respectively. The EDX results reveal the relative amounts of copper, iron, oxygen and sulphur in the material that are tabulated in Table S1 and S2 for pristine CuFe_2_O_4_ and S-CuFe_2_O_4_ photocatalysts respectively. Results have confirmed the purity and existence of sulphur dopants in CuFe_2_O_4_ photocatalysts.

### UV–VIS-DRS

**Figure 5:**
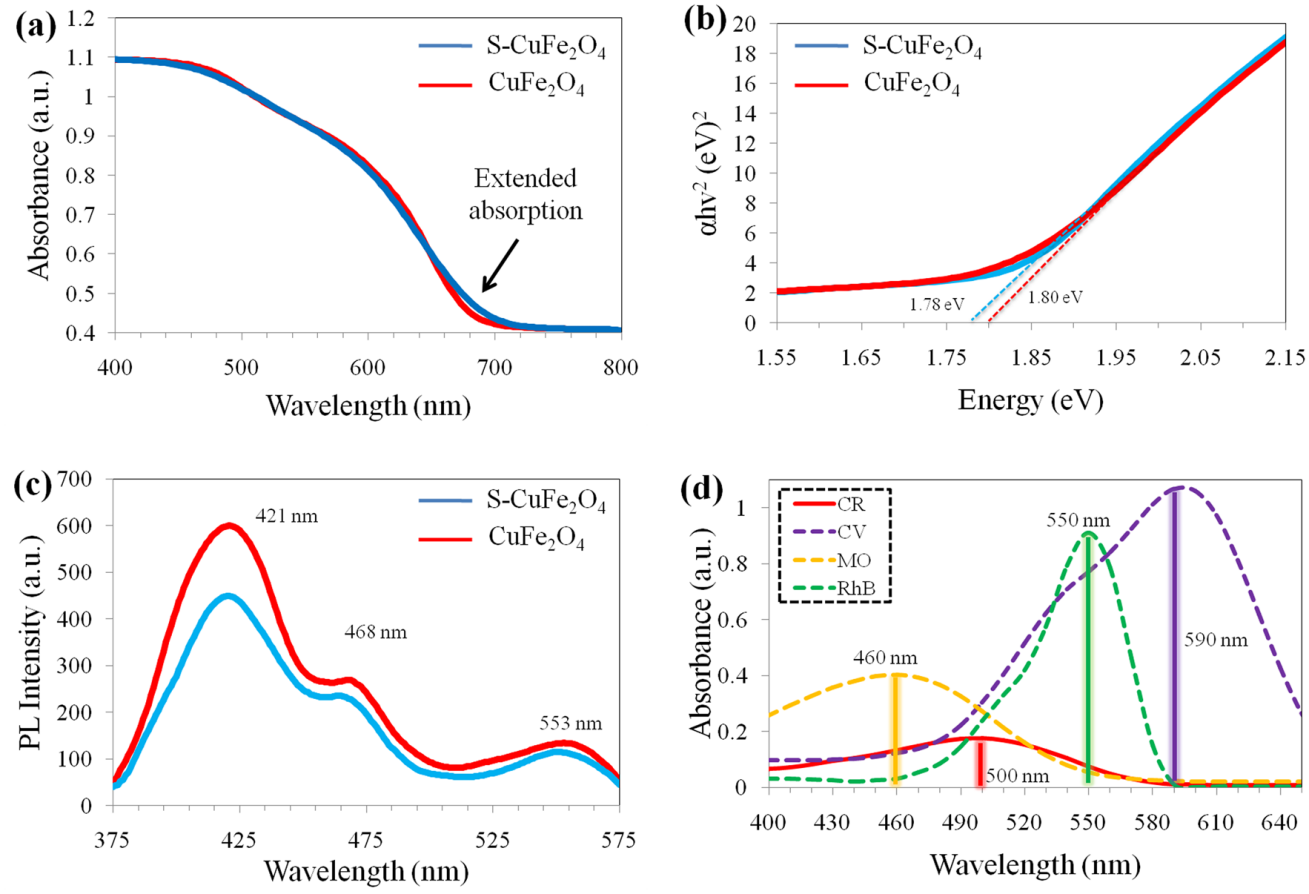
(**a**) UV–Vis-DRS (**b**) Band gap measurements for allowed transitions (**c**) PL results of CuFe_2_O_4_ and S-CuFe_2_O_4_where (**d**) represents the λ_max_ of CR, CV, MO and RhB dyes.

The optical properties of pristine CuFe_2_O_4_ and S-CuFe_2_O_4_ were investigated by their UV–Vis-DRS study. The UV–Vis-DRS has advantage over UV–Vis absorption spectroscopy specifically to obtain the optical properties of powdered sample because it has less scattering effects than the absorption of liquids^52^. Figure 5a illustrates an absorption edge that indicates the Urbach resemblance absorption tail within visible region^53,54^. CuFe_2_O_4_ shows maximum absorption at ~ 688 nm, corresponding to its optical band gap i.e. Eg = 1.80 eV. Sulphur doping slightly red-shifted the visible absorption to around ~ 696 nm (Eg = 1.78 eV), showing excellent visible light response which results in higher photocatalytic activities, the band gap energy plot showed in Fig. 5b. The optimal band gap of pure and doped CuFe_2_O_4_ was determined by Eq. (3).

3 $${\left(\alpha h\nu \right)}^{2}= A (h\nu -{E}_{g})$$

### PL analysis

The photoluminescence (PL) of as-synthesized photocatalysts provides the fundamental information to determine the photo-induced electron–hole (e^‒^/h^+^) pairs to trap and transfer processes. Figure 5c represents the comparative results for CuFe_2_O_4_ and S-CuFe_2_O_4_ which exhibit the broad peaks at 421 nm, 468 nm and 553 nm^55^. The decrease in the intensity in case of S-CuFe_2_O_4_ indicates the sulphur incorporation^56^. Moreover, this decrease intensity evidenced a greater transfer of electrons to active centers. PL peak at 468 nm corresponding to blue emission is due to imperative defects associated with the interface traps existing at the grain boundaries. Similarly, the peak at 553 nm corresponds to defect sites of oxygen vacancies. In S-CuFe_2_O_4_ emission is slightly shifted to 549 nm, attributed to the appearance of electronic levels of intrinsic characteristics^57^. It specifies that back recombination of electrons and holes are remarkably lower in case of S-CuFe_2_O_4_ catalysts. Due to doping of sulphur, several mid-gap states are formed between valance bands as well as in conduction bands within the structures of prepared catalysts. These mid-gap energy states result in electron/hole pair separation successively because sulphur serves as a charge-separating center^58^. In PL spectroscopy, a broader peak reveals better electron–hole separation, while high intensity peaks exhibit fast electron–hole recombination^59^. It is obvious from the PL spectra that S-CuFe_2_O_4_ display highly enhanced photocatalytic performance due to an efficient charge carrier separation^60^.

### Dye degradation studies

**Figure 6:**
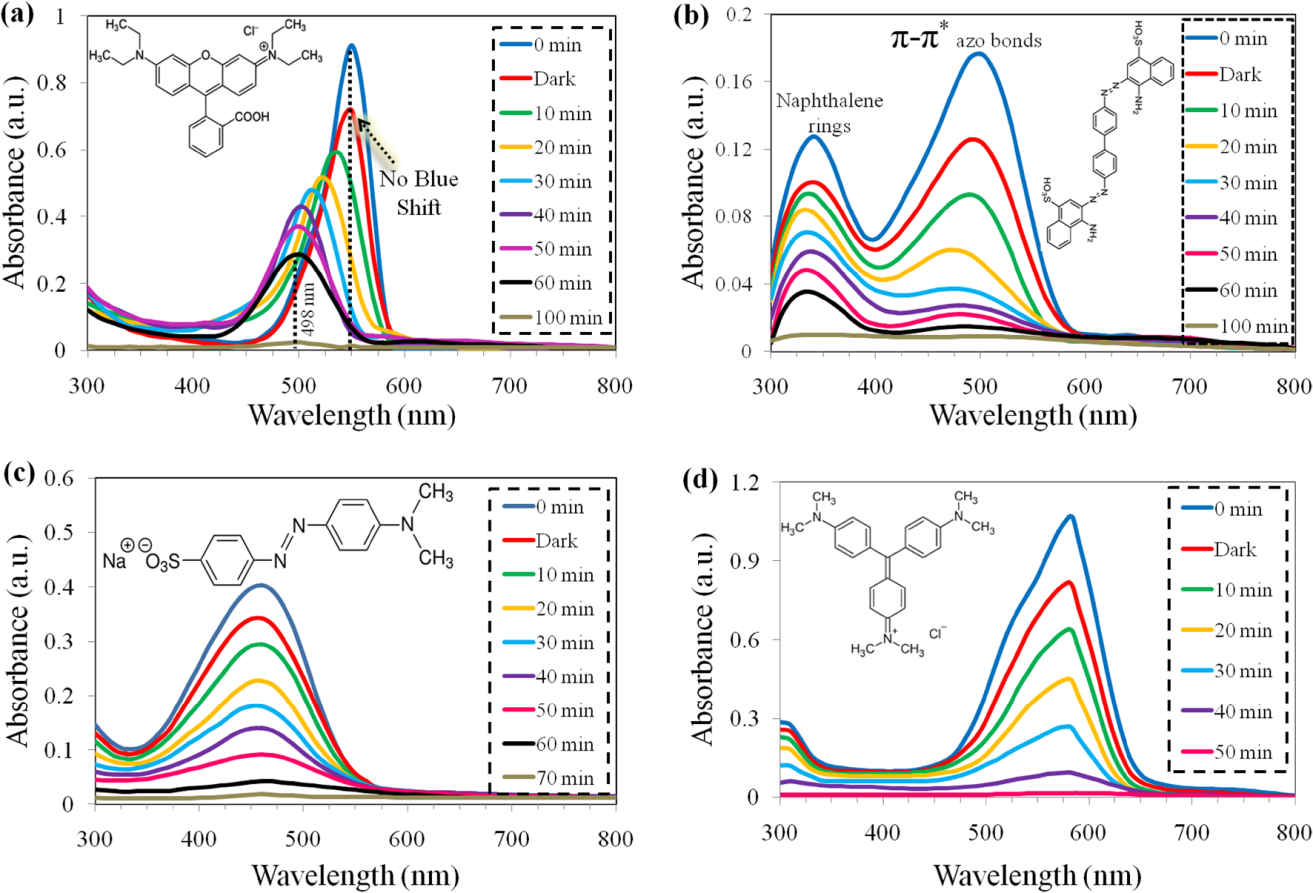
(**a**) RhB, (**b**) CR, (**c**) MO and (**d**) CV degradation results.

**Figure 7:**
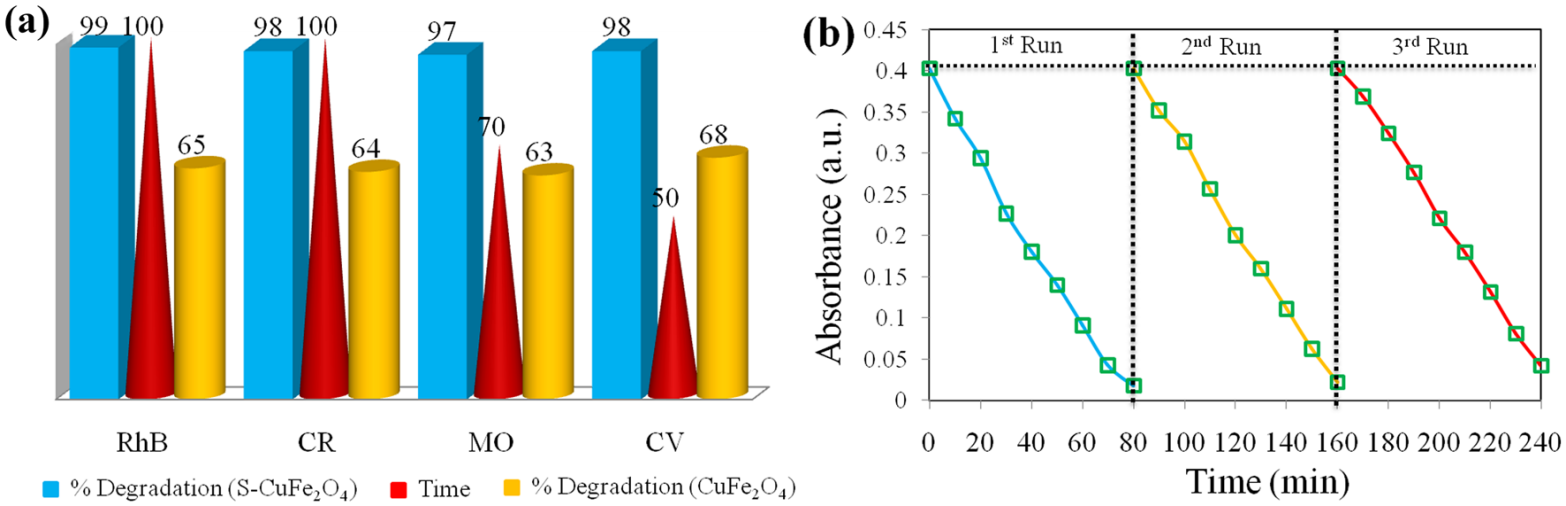
(**a**) Comparison of % degradation of RhB, CR, MO and CV using pristine (yellow) and S-doped CuFe_2_O_4_ (blue) photocatalysts and (**b**) recyclability tests of S-CuFe_2_O_4_ photocatalysts for MO degradation.

Figure 5d shows lambda max (λ-max) values for selective four toxic dyes such as CV, RhB, CR and MO having wavelength maxima at 590, 550, 500 and 460 nm respectively. The basic information and structure of selected dyes are demonstrateds in supporting information. The degradation results of aforementioned dyes over as-prepared photocatalysts were monitored at different time intervals in 300‒800 nm range. The results for the removal of RhB shown in Fig. 6a, indicate that S-CuFe_2_O_4_ photocatalysts adsorb 20.2% (302 mg/g) dye in the absence of visible light (dark). This adsorptive removal is accomplished without change in their λ-max. The amount of adsorbed dye at equilibrium q_e_ (mg/g) was calculated by using Eq. (4):

4 $$qe=(Co-Ce)V/m$$

where m is catalyst weight, V (L) is volume of solution and Co and Ce are concentrations at initial and equilibrium state of RhB solution^61^. Then sample was irradiated with visible light to examine the photocatalytic efficiency of the S-CuFe_2_O_4_. After 100 min, the concentration of degraded dye reached to ~ 99%. Comparison of degradation efficiency of pristine CuFe_2_O_4_ with S-CuFe_2_O_4_ shows 34% difference, see Fig. 7a. On the basis of PL and UV–VIS spectrophotometric studies, it could be concluded that presence of sulphur has enhanced the photocatalytic efficiency of copper ferrites by decreasing the electron hole recombination^47^. Rhodamine B has one carboxylic acid (‒COOH) and two amino groups (‒NH_2_). Ethyl molecules are electron promoters and donate electron to xanthene rings, as a result shifting the λ_max_ to higher wavelength^62^. The higher λ_max_ is due to electron donating effect of ethylene molecules. During the photoreaction, ethylene molecules are removed (de-ethylation), due to which the absorbance shifts towards lower wavelength called blue shift. It is observed that λ_max_ in the graph shifted from 550 to 498 nm. It has been obvious that, blue shift may arise due to energy difference between HOMO and LUMO when rhodamine B is grafted with S-CuFe_2_O_4_ catalysts. HOMO being filled; its electrons get attracted to the polar S-CuFe_2_O_4_ by weak dipole–dipole interactions. This attraction lowers the energy of HOMO while empty LUMO energy states of catalysts remain unaffected. This transition shifts the λ_max_ to the lower wavelength^63^. Besides this, the appearance of blue shift is due to the step by step N-de-ethylation^64^. This de-ethylation gives rise to N,N,N-triethylrhodamine, N,N-diethyl rhodamine and N-ethyl rhodamine. The final photo-degraded product is assumed to be a completely de-ethylated compound (rhodamine) that has characteristic absorbance maximum at 498 nm^65^.

Figure 6b represents absorption spectra of Congo red (CR) at different time intervals. Photoreaction of CR dye solution by S-CuFe_2_O_4_ photocatalysts starts after it attains equilibrium between adsorption and desorption in the dark (no light). This adsorption of dye in dark is due to the attraction of two oxygen atoms of sulfonate groups (CR dye) with surfaces of catalysts^65^. This removal is accomplished by parallel decrease in intensity of both peaks without change in their λ_max,_ that is 340 nm for naphthalene rings and 500 nm for π-π* azo bonds^66^. After that, it was irradiated with visible light in order to initiate photocatalytic degradation. The absorption spectrum of CR shows two distinct peaks; peak at 500 nm is due to π-π* azo bonds and second peak at 340 nm is due to naphthalene rings. Actually, π-π* azo bonds are degraded easily as compared to naphthalene ring. After 100 min, the dye is almost completely removed due to destruction of its chromophoric structure in the vicinity of azo-linkages^67^.

Figure 6c represents absorption spectra of methyl orange (MO). The UV–vis spectra of methyl orange have a distinct broad band in the visible region, with maximum absorption at 460 nm due to the chromophore structure of the dye^68^. It is clear from results that after 70 min, the dye has degraded as a result of photoreaction with S-CuFe_2_O_4_ photocatalysts. This is due to the destruction of homo- and hetero polyaromatic rings present in methyl orange^69^. It has been investigated that during photoreaction, the –N=N– double bond breaks and simple phenyl rings are formed with amine groups as degraded products^70^.

Crystal violet (CV) degradation is exhibited in Fig. 6d which represents a sharp peak at about 590 nm. Previously reported studies explain that the degradation of crystal violet dye can take place via different mechanisms such as N-demethylation, chromophore cleavage and breaking in ring structure^71^. The degradation mechanism and pathway depends on the type of catalysts, degradation method and oxidizing agents used^72^. After 50 min, the violet color of dye solution had completely faded away. The comparative efficiencies of reported ferrites as-well as-prepared S-CuFe_2_O_4 _are illustrated in the Table 2.

**Table 2:** Comparison of photocatalysts used for the degradation of dyes.

Catalyst	Dye	Degradation efficiency (%)	Degradation time (min)	Light source	References
CuFe_2_O_4_	MO	53	90	UV light	^73^
Ce-doped CuFe_2_O_4_	MO	66	60	UV light	^74^
Sm-doped CuFe_2_O_4_	MO	66	60	UV light	^75^
CoFe_2_O_4_	MO	82	90	UV light	^76^
BiFeO_3_	MO	86	150	Sunlight	^77^
S-CuFe_2_O_4_	MO	97	70	Visible light	Current work
C-CuFe_2_O_4_	RhB	30.5	360	Visible light	^78^
C-CuFe_2_O_4_/ZnO	RhB	86.9	360	Visible light	^78^
NiFe_2_O_4_@HAp-Sn^2+^	RhB	84.45	60	Sunlight	^79^
S-CuFe_2_O_4_	RhB	99	100	Visible light	Current work
NiFe_2_O_4_	CR	96.80	60	Visible light	^80^
Mg-Co ferrite	CR	95.40	150	visible light	^81^
Fe_3_O_4_	CR	77	60	Visible light	^82^
Cts/Fe_3_O_4_	CR	98	60	Visible light	^82^
S-CuFe_2_O_4_	CR	98	100	Visible light	Current work
MGF_3_	CV	97.67	60	Sunlight	^83^
BaFe_2_O_4_	CV	90	10	Microwave	^84^
KFe_4_O_7_	CV	92	35	Visible light	^85^
S-CuFe_2_O_4_	CV	98	50	Visible light	Current work

### Comparison and recyclability

Figure 7a illustrates the degradation efficiencies of S-doped CuFe_2_O_4_ in comparison with pristine CuFe_2_O_4_ for RhB, CR, MO and CV. After 100 min, 99% RhB was degraded by S-doped CuFe_2_O_4_ but pristine CuFe_2_O_4_ was able to degrade only 65% of it. Similarly CR was degraded to 98% by S-doped CuFe_2_O_4_ and 64% by pristine CuFe_2_O_4_ when measured after 100 min. 97% of MO was degraded by S-doped CuFe_2_O_4_ and 63% degradation occured with pristine CuFe_2_O_4_ after 70 min. For CV, 98% of dye degradation occured in only 50 min by S-doped CuFe_2_O_4_ and 68% in presence of pristine CuFe_2_O_4_. It could be concluded that S-CuFe_2_O_4_ nanostructures are more efficient photocatalysts as compared to the pristine CuFe_2_O_4_ because sulphur atoms introduce new active sites that enhance the interactions with the adsorbed dye molecules. Moreover, sulphur dopants modify the electronic structure of the photocatalysts, leading to changes in the bandgap and energy levels. This improves the separation of electron–hole pairs and reduces recombination, leading to more efficient use of the generated charge carriers for dye degradation. Another advantage associated with the use of S-CuFe_2_O_4_ photocatalysts is its good magnetic properties that allows it to be easily separated from reaction mixture by using external magnet. This separation technique is much easier, faster and efficient as compared to other conventional techniques such as filtration and centrifugation^86^. This also eliminates the risk of secondary pollution from catalysts as well as its full utilization for long times. The recyclability tests were performed for each cycle after careful washing and drying. Figure 7b shows recyclability results of S-CuFe_2_O_4_ for MO degradation. After being used three times for the degradation of MO, a slight difference in catalyst’s efficiency was observed due to loss of catalyst during recovery process.

### Mechanism of dye degradation

**Figure 8:**
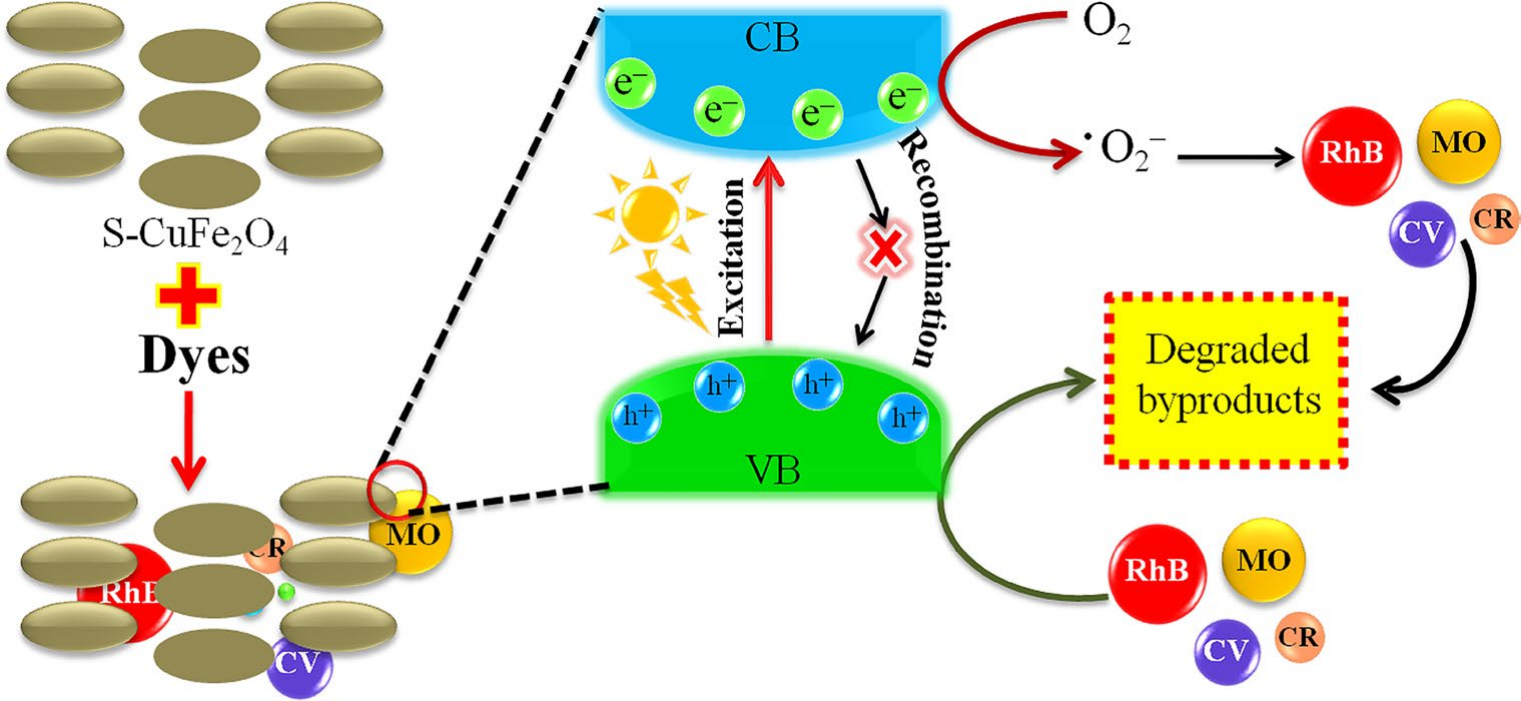
Photocatalytic dye degradation reaction over S-CuFe_2_O_4_ photocatalysts.

**Scheme 1:**
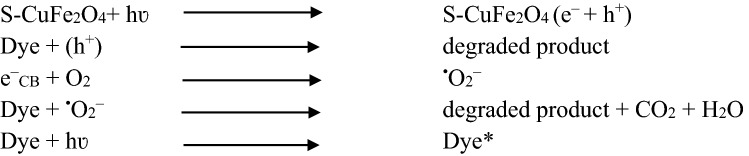
Photocatalytic reactions involved in dye degradation.

The degradation mechanism of MO, RhB, CV and CR dyes are shown in Fig. 8. In absence of light, dyes are adsorbed on the surface of photocatalysts. When visible light is irradiated, photoreaction is governed by the excitation of electrons and holes. The electrons start to migrate from valance band to conduction band of photocatalysts. The available electrons at the conduction bands levels readily react with oxygen to generate superoxide radicals (^⋅^O_2_^‒^). These superoxide radicals (^⋅^O_2_^‒^) react with dyes and convert them to degraded byproducts^87^. At the same time, holes available in valance band levels of catalysts oxidize the dyes over the surfaces of catalysts. Presence of sulphur in the catalysts not only captures electrons but also prevents the recombination of charges (back reaction) and also can produce superoxide radicals (^⋅^O_2_^‒^) over active sites of catalyst by transporting electrons to oxygen^47^. Moreover, holes oxidize the H_2_O molecules into ^˖^OH radicals, these hydroxyl radicals further enhance the dye adsorption at the surfaces of catalysts^88,89^. Due to higher dye adsorption at catalysts surfaces, rate of dye degradation is readily increased (Scheme 1).

## Conclusion

In this work, CuFe_2_O_4_ and S-CuFe_2_O_4_ photocatalysts were successfully synthesized by employing hydrothermal methods. For structural morphologies XRD, Raman and FT-IR techniques were subjected to confirm crystallinity and sulphur doping. The results depicts that the doping of non-metallic anionic sulphur causes strain in the lattice and enter the lattice as anions by replacing oxygen. SEM results confirm that sulphur doping reduces the agglomeration and increases the actives cites for photoreactions. EDX confirms the presence of sulphur whereas TGA confirms the stability of as-synthesised S-CuFe_2_O_4_ at higher temperatures. PL results gave evidences that sulphur was capable to act as electron trapping centre which suppressed the recombination of charges during the photoreaction. The CuFe_2_O_4_ and S-CuFe_2_O_4_ photocatalysts were used to degrade RhB, CR, MO and CV dyes, results revealed that doping of sulphur boosts the photocatalytic degradation reactions. It has been found that by using S-CuFe_2_O_4_, RhB degradation efficiency has been increased from 65 to 99% within 100 min. The dye degradation results of this work assure the superior performances of S-CuFe_2_O_4_ than pristine CuFe_2_O_4_. On the basis of photocatalytic efficiencies, this work can be assigned as excellent candidate for photocatalysis applications.

## Supplementary Information

Supplementary Information.

## Data Availability

All data used and analyzed in current work are included in this article and SI file.
