# Supplementary material for: Tunable sulphur doping on CuFe2O4 nanostructures for the selective elimination of organic dyes from water
Source: Sci Rep. 2023 Apr 18;13:6306. doi: 10.1038/s41598-023-33185-0 (PMC10113332; doi:10.1038/s41598-023-33185-0)
Supplement: Supplementary file 1 — Supplementary Information. [file 41598_2023_33185_MOESM1_ESM.docx]

**Supporting Information**

**Tuneable Sulphur Doping on CuFe_2_O_4_ Nanostructures for the Selective Elimination of Organic Dyes from Water**

Anam Aslam^a^, Muhammad Zeeshan Abid^a^, Khezina Rafiq^a^, Abdul Rauf^a^, Ejaz Hussain^a^**^*^**

^a^Institute of Chemistry, Inorganic Materials Laboratory 52S, The Islamia University of Bahawalpur-63100, Pakistan

**
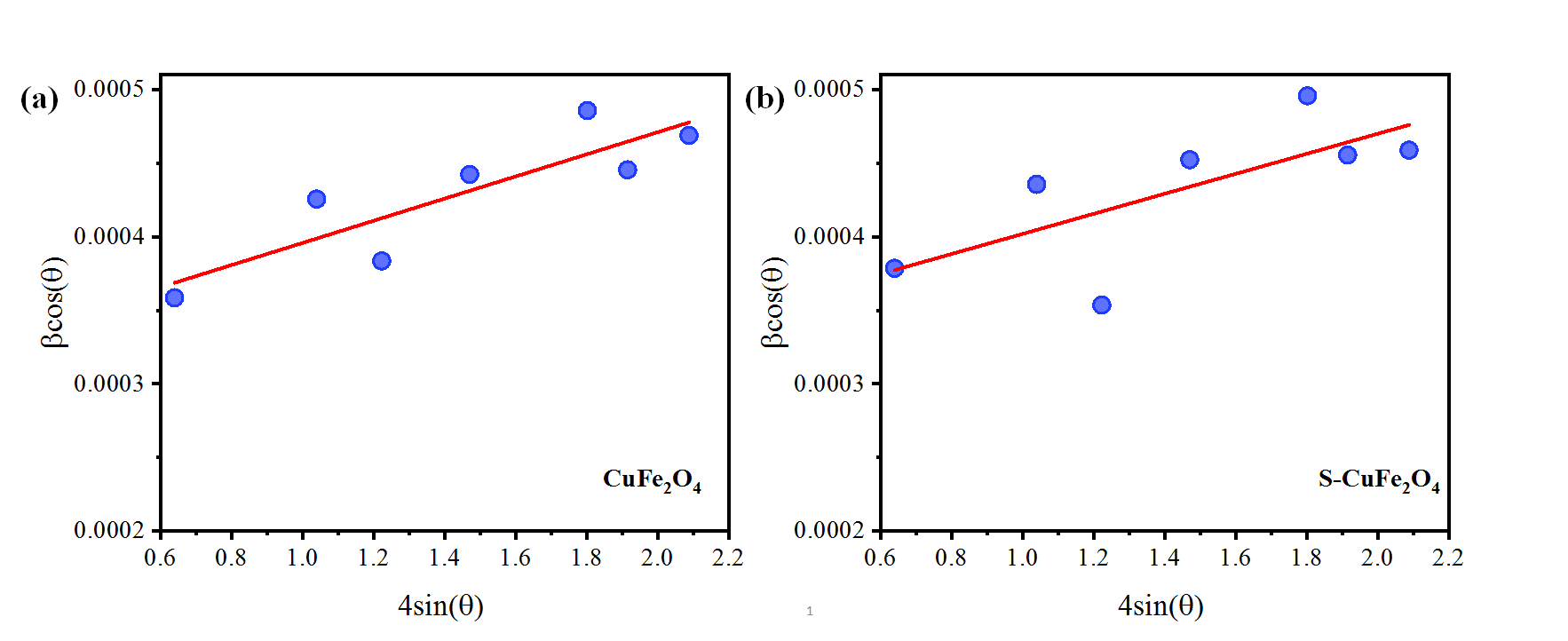
**

**Figure S1**: Williamson-Hall plots for size calculation (a) CuFe_2_O_4_ and (b) S-CuFe_2_O_4_.

**Table S1:** Weight and Atomic percentage (EDX Analysis) of elements in as-synthesized CuFe_2_O_4_.

| **Elements** | **Weight %** | **At %** |
| --- | --- | --- |
| C | 0.65 | 1.69 |
| O | 26.29 | 55.74 |
| Fe | 46.91 | 28.55 |
| Cu | 26.15 | 14.02 |
| Totals | 100 | 100 |

**Table S2:** Weight and Atomic percentage (EDX Analysis) of elements in as-synthesized S-CuFe_2_O_4_.

| **Elements** | **Weight %** | **At %** |
| --- | --- | --- |
| C | 0.60 | 1.67 |
| O | 26.89 | 56.24 |
| S | 1.2 | 1.23 |
| Fe | 46.96 | 28.13 |
| Cu | 24.35 | 12.73 |
| Totals | 100 | 100 |

**Preparation of solutions:** 0.1 M copper nitrate solution was prepared by dissolving 2.96g of Cu(NO_3_)_2_.6H_2_O (Sigma Aldrich, 99%) in 30ml of deionized water. 0.2M iron nitrate solution was prepared by dissolving 1.104g of Fe(NO_3_)_3_.9H_2_O (Sigma Aldrich, 99%) in 30ml of deionized water. 0.01 M thiourea solution was prepared by dissolving 0.76g of CH_4_N_2_S (Sigma Aldrich, 98%) in 30ml of distilled water. 0.01 M glucose solution was prepared by dissolving 0.018g glucose (Sigma Aldrich, 99.5%) in 30ml deionized water under magnetic stirring.

**Structure of CV dye:** Crystal violet or gentian triarylmethane is a cationic dye. It is also known as hexa methyl pararos aniline chloride or methyl violet 10B. The molecular formula of CV is C_25_H_30_ClN_3_ and molecular weight is 407.98. The melting point of crystal violet is 205 °C (478 K or 401 °F) [1]. The molecular structure of dye is shown in Figure S2:

Figure S2: Structure of CV dye.

**Structure of MO dye:** Methyl orange (MO) is an anionic azo dye. Its chemical name is dimethyl amino azo benzene sulfonate having molecular formula C_14_H_14_N_3_NaO_3_S. Molecular weight and density of MO dye is 327.33g/mol and 1.28g/cm^3^ respectively. Melting point of methyl orange is ˃300℃. It contains aromatic and –N = N– groups in its molecule, which are highly toxic and carcinogenic [2].

Figure S3: Structure of methyl orange.*

**Structure of RhB dye:** Rhodamine blue is a cationic dye having molecular formula C_28_H_31_ClN_2_O_3_ and molecular weight 364.4 g/mol. Melting point of rhodamine blue is 210 ℃ (410 ℉ and 483 K) [3].

Figure S4: Chemical structure of RhB

**Structure of CR dye:** Congo red is an anionic dye presenting two azo bonds (-N=N-) chromophore. Its molecular formula is C_32_H_22_N_6_Na_2_O_6_S_2_ with a molecular weight 696.66 g/mol and melting point ˃360℃. It is highly soluble in water [4].

Figure S5: Structure of Congo red dye.

**References:**

1. Mittal, A., et al., *Adsorption of hazardous dye crystal violet from wastewater by waste materials.* Journal of colloid and interface science, 2010. **343**(2): p. 463-473.

2. Jamil, T.S., et al., *Enhancement of TiO2 behavior on photocatalytic oxidation of MO dye using TiO2/AC under visible irradiation and sunlight radiation.* Separation and purification technology, 2012. **98**: p. 270-279.

3. Waghchaure, R.H., V.A. Adole, and B.S. Jagdale, *Photocatalytic Degradation of Methylene Blue, Rhodamine B, Methyl orange and Eriochrome Black T Dyes by Modified ZnO Nanocatalysts: A Concise Review.* Inorganic Chemistry Communications, 2022: p. 109764.

4. Liu, J., et al., *Adsorption of Congo red dye on FexCo3-xO4 nanoparticles.* Journal of environmental management, 2019. **238**: p. 473-483.
